# Psychology of personal data donation

**DOI:** 10.1371/journal.pone.0224240

**Published:** 2019-11-20

**Authors:** Anya Skatova, James Goulding

**Affiliations:** 1 School of Psychological Science, University of Bristol, Bristol, England, United Kingdom; 2 Horizon Digital Economy Research, University of Nottingham, Nottingham, England, United Kingdom; 3 Alan Turing Institute, London, England, United Kingdom; 4 N/lab, Nottingham Business School, University of Nottingham, Nottingham, England, United Kingdom; Middlesex University, UNITED KINGDOM

## Abstract

Advances in digital technology have led to large amounts of personal data being recorded and retained by industry, constituting an invaluable asset to private organizations. The implementation of the General Data Protection Regulation in the EU, including the UK, fundamentally reshaped how data is handled across every sector. It enables the general public to access data collected about them by organisations, opening up the possibility of this data being used for research that benefits the public themselves; for example, to uncover lifestyle causes of poor health outcomes. A significant barrier for using this commercial data for academic research, however, is the lack of publicly acceptable research frameworks. Data donation—the act of an individual actively consenting to donate their personal data for research—could enable the use of commercial data for the benefit of society. However, it is not clear which motives, if any, would drive people to donate their personal data for this purpose. In this paper we present the results of a large-scale survey (N = 1,300) that studied intentions and reasons to donate personal data. We found that over half of individuals are willing to donate their personal data for research that could benefit the wider general public. We identified three distinct reasons to donate personal data: an opportunity to achieve self-benefit, social duty, and the need to understand the purpose of data donation. We developed a questionnaire to measure those three reasons and provided further evidence on the validity of the scales. Our results demonstrate that these reasons predict people’s intentions to donate personal data over and above generic altruistic motives. We show that a *social duty* is the strongest predictor of the intention to donate personal data, while *understanding the purpose of data donation* also positively predicts the intentions to donate personal data. In contrast, *self-serving motives* show a negative association with intentions to donate personal data. The findings presented here examine people’s reasons for data donation to help inform the ethical use of commercially collected personal data for academic research for public good.

## Introduction

As we go about our daily routines, we leave a trail of digital information which is harvested and harnessed by industry to optimise their services and increase profits. Retailers have used this data to revolutionise their working practices by leveraging personal data streams for business processes and customer experience optimisation [1–3]. Attention has turned to the value that these everyday digital data streams, representing real-world and real-time behaviours, could contribute to benefit the public good by being used for health research [4–7]. Examples include mobile phone data which encode patterns of mobility, isolation, physical activity and sleep; retail data revealing calorie and nutrient intake [8], medication adherence and alcohol consumption; transport data that can evidence our lifestyles and daily contexts; web logs reflecting the issues that concern us the most [9].

Historically using industry data for academic health research has been difficult due to the absence of established frameworks for personal data sharing between industry and non-industry (e.g., academic) researchers, and the absence of choice for individuals to decide with whom to share their data. Previously, whilst individuals generated personal data reflecting various aspects of their behaviour which had value for companies, they had little power over how their data was used, in the best-case scenario trading benefits for the use of their data; for example, shoppers use loyalty cards to receive discounts in their purchases. Changes in data sharing and privacy laws, such as the introduction of General Data Protection Regulation (GDPR) drastically altered the rights individuals in the EU have about personal data collected on them [10]. Specifically, GDPR introduces Right to Data Portability, which allows data subjects (i.e., general public) request to obtain data that a data controller (i.e., supermarket) holds on them and to reuse it for their own purposes. Because of these changes in data law, it is now possible for the industry-collected data to be shared by individuals for research benefiting public good as individuals are free to either store the data for personal use or to transmit it to another data controller (i.e., academic researcher). The right to data portability applies to personal data that an individual has given to a data controller; when the processing is carried out by automated means and includes observed data about the individual (e.g., person’s search history or record of their shopping). This brings with it the potential of opening up vast untapped pre-existing data resources that could advance health research as it is now possible for a member of the general public to transfer their personal data (collected by any commercial entity) to an academic researcher in a machine-readable format. Some limitations of Right to Data Portability include timescales–a data controller can take up to a month for the data transfer, and the fact that this right does not include inferred data on the individual that a data controller might have (e.g., political interests or sexuality inferred from online search history).

Whilst such data transfer is theoretically possible, the questions remain; would people donate their personal data for academic health research, and if so, what would motivate them to engage in this prosocial act? To explore these questions, here we outline the similarities of data donation with other prosocial behaviours, and examine how different prosocial motivations could manifest themselves in acts of data donation.

### Data donation and prosocial behaviour

The advent of the digital economy and an increasingly digitally connected society has produced a spectrum of new sharing relationships. These range from food sharing [11], to crowdfunding [12], mass digital fundraising [13] and slacktivism [14,15]. Some of these activities are characterised as ‘digital philanthropy’: the process of donating various types and forms of data by companies for public good [16,17].

‘Prosocial behaviour’ is an umbrella term that describes activities undertaken to benefit other individuals or society as a whole [18], including actions such as volunteer work [19], helpful interventions [20]; blood donation [21] and donating money to those in need [22]. Millions of people around the globe regularly volunteer for such endeavours which benefit society.

Donating personal data, similar to the way we donate blood, could become a new act of digital economy prosocial behaviour. If ways can be found to encourage and enable individuals to donate their ‘digital footprint’ for academic research, this could contribute to knowledge in many domains. Prosocial behaviours are a common and valuable part of society, and data donation would likely encompass the generic features of these prosocial behaviours [23]. Further, research shows that different motivations incentivise individuals to behave prosocially. To understand what the specific catalysts for data donation could be, we outline what is known about the factors that are associated with motivation(s) to engage in prosocial behaviours.

### Prosocial motivations and behaviours

The psychological factors behind prosocial behaviours are well studied [24–26]. The cluster of prosocial motivations [27] is one of the main predictors of ‘intention to donate’ and help others in many domains (e.g., blood donation, [28]). Recent research has demonstrated the multifaceted nature of prosocial motivation (e.g., in the domain of blood donation, [29]), which is important in understanding how to encourage different people to engage in prosocial behaviours. For example, again in the area of blood donation, campaigns often focus on altruistic motivation (e.g., “Donate blood. Save a life”). However, other motivations can also affect the decision to donate (e.g., “warm glow”, [30,31]). Understanding differences in prosocial motivations to donate personal data therefore has implications for the efficacy of campaigns encouraging the sharing of personal data to benefit society. To explore this issue, we developed a questionnaire to measure reasons to donate person data. Below we discuss prosocial motivation literature and various prosocial behaviour motivating factors, which were used to develop our questionnaire to measure reasons for data donation.

*Altruism* is defined as a desire to help others with no explicit benefit to the self [32,33]. Altruism, and its forms, are the most common constructs linked to costly prosocial behaviour, such as blood or bone marrow donation [34]. In addition to pure altruism, previous research has identified *reluctant altruism*; donating because no-one else would [29]. Further, behavioural economics literature suggested another form of altruism, the *warm glow*, which is shown to be a central driver for charitable donations. *Warm glow* combines the desire to help as well as expecting to feel positive after the act of helping [35,36]. It has been shown that *warm glow* was the only positive predictor of intentions to donate blood in a donors’ sample [28,36].

*Self-benefiting motivations*, including self-regarding motives (e.g., receiving gifts in return for donation, being able to put donation experience on the CV) or another form of warm glow, *egalitarian warm glow* (e.g., feeling good about yourself after donating) have also shown to motivate prosocial behaviour in the domains like blood donation [29].

An important driver for prosocial behaviour is *social responsibility*, or the feeling of duty. It relates to indirect reciprocity: giving back to the community and expecting the same treatment in return [37,38]. Steele et al [39] showed that social responsibility was higher in people who continued to donate blood, as compared to those who lapsed. Further, research has shown that when the feeling of duty was experimentally induced, it increased the frequency of actions to help others [40].

*Emotional* states have been also been shown to predict prosocial behaviour [41]. Specifically, negative emotions, such as guilt, have been shown to increase likelihood to cooperate [42] and help someone in need [43,44]. Research has shown that negative emotions elicited by charities’ marketing campaigns enhanced their effectiveness [45]. If guilt is manipulated, participants offer more help and demonstrate higher levels of prosocial behaviour [46]. In blood donation literature, avoiding negative emotions by the act of donation increases intentions to donate [47].

Whilst there is extensive research focusing on the motivations for prosocial behaviour in general [27], and in specific domains (e.g., blood donation), there is only preliminary research on the different reason(s) to donate personal data. Skatova et al [48] showed that two main groups of motives are associated with the intention to donate personal data: social responsibility, and self-serving interests. The current research builds on this work and studies which other aspects of prosocial motivation could drive personal data donation.

### The study

As ‘intention to act’ is a key factor in determining blood donation behaviour [49–53], we focused on the ‘intention to donate personal data’ as the first step in studying data donation behaviour and whether individuals find donating personal data feasible and acceptable. The second aim of the paper was to study psychological factors associated with intention to donate personal data based on the wealth of literature on prosocial motivations.

Of the existing validated psychometric tools that could be used to assess motivations to donate (e.g., Prosocial Tendencies Measure, [54]), none measure reasons to donate personal data directly. It has been shown that domain specific indices of motivation predict real world and intended behaviour better than generic motives [29,55]]. Thus, to address the second aim of this paper, we developed and tested psychometric properties of a *Reasons for Data Donation* questionnaire.

Based on previous literature and previous preliminary research [48] we developed questions reflecting different reasons why individuals may choose to donate their personal data around three factors (see S1 Table for the full list of items): (1) social duty, including both reluctant and impure altruism, as well as social responsibility; (2) self-serving motives, including egalitarian warm glow, self-regarding motives; and (3) motives related to the avoidance of negative feelings, and specifically guilt.

In addition to prosocial motives related to social duty, self-regarding motives and avoidance of negative feelings, we included items reflecting the need to understand how personal data would be used after it had been donated. Cognitive attitudes, or weighting pros and cons of the decision to donate have been shown to positively predict intentions to donate blood [56]. Understanding why and how personal data will be used, as well how the act of data donation could affect society are potentially important factors affecting the decision to donate personal data.

To assess the psychometric properties of the newly developed questionnaire, we studied convergent and incremental validity of the Reasons for Data Donation questionnaire. To demonstrate convergent validity, we used previous instruments that have been associated with prosocial motivations: Prosocial Tendencies Measure [54], Self-Report Altruism Scale [57], Interpersonal Reactivity Index [58] and the Big Five questionnaire [59]. Prosocial Tendencies Measure provides a measure of different drivers of prosocial behaviour, and includes five scales: Public, Anonymous, Dire, Emotional, and Compliant. Conceptually, the Public Prosocial Tendencies Measure scale should be associated with self-regarding motives; while Dire and Emotional Prosocial Tendencies Measure scale with avoidance of negative feelings. Self-Report Altruism Scale is an index of previous prosocial behaviours in a variety of domains and we expected that the intention to donate, the social duty factor, as well as avoidance of negative feelings items would be associated with Self-Report Altruism Scale score. Finally, Interpersonal Reactivity Index scale measures trait empathy which is an important factor in regulating prosocial behaviour [60,61], with prosocial motivation being strongly linked to individual differences in empathy [62]. Interpersonal Reactivity Index includes four subscales: Empathic Concern, Perspective-Taking, Fantasy and Personal Distress. We expected the social duty factor to be associated with Perspective Taking and Empathic Concern, as it had previously in other domains [63], while avoidance of negative feelings items to be conceptually associated with Personal Distress, and we expected avoidance of negative feelings items and Personal Distress to be positively associated. Finally, we measured general personality traits, such as Agreeableness and Extraversion from the Big Five model, because they have been shown to be associated with prosocial behaviour [64,65]. We expected the social duty factor to be associated with Extraversion and Agreeableness, and the cognitive attitudes factor to be associated with Openness to New Experiences; another facet of the Big Five.

To demonstrate incremental validity of the new scales, we tested whether they predicted the intended decision to donate over the Prosocial Tendencies Measure, and the Self-Report Altruism Scale. Both Prosocial Tendencies Measure and Self-Report Altruism Scale reflect more generic motives and do not measure specific reasons behind particular choices, such as the intention to donate personal data. It is plausible that a person is generally community oriented and wishes to help others, but they might not intend to donate personal data because they do not understand how it will be used in the future. Thus, we expected that reason for data donation scales to positively correlate with respective Prosocial Tendencies Measure and Self-Report Altruism Scale scales, but the new measure of motivation to donate personal data to predict the intention to donate above general prosocial motivation scales.

## Method

### Materials

The Reasons for Data Donation scale contained 30 items querying attitudes towards data donation, for example “*I would feel positive after donating my loyalty card(s) data to health research organisation/charity*.” Each item was scored on a five-point Likert scale from “strongly disagree” to “strongly agree”. The items for the Reasons for Data Donation were designed based on the literature discussed in the Introduction and previous pilot work [48], see S1 Table for the full list of items as well as which constructs each item was based on. Descriptive information on each item is presented in the Results section.

The Prosocial Tendencies Measure [54] is a 23-item self-report measure. Each item is a phrase for which the participant is asked to give an indication of how well the item describes them; an example phrase is *“I believe I should receive more recognition for the time and energy I spend on charity work*.” Participants are asked to score themselves on a 5-point Likert scale ranging from “does not describe me at all” to “describes me greatly” for each item. The scale is scored by averaging the answers to each item within a subscale providing a minimum score of 1 and a maximum score of 5. Analysis of the subscales, along with the means, standard deviations and Cronbach’s alphas are presented in the Results section.

The Self-Report Altruism Scale [57] is a one factor 20-item scale. For each item, participants needed to indicate the regularity of past behaviour on a 5-point Likert scale from never (0) to very often (4); a final score is produced by summing the value of the items. An example item for the Self-Report Altruism Scale is “*I have given directions to a stranger*.” Due to limitations in space, we administered only the 14 most relevant items to the study. Items that were not used were either repetitive (e.g.,”*I have given money to a charity”*, was not used, whereas *“I have donated goods or clothes to a charity”*, was used) or outdated/not relevant to the context of the study, which took place in the UK (e.g., “*I have helped push a stranger’s car out of the snow*”). The scale showed high internal consistency: α = .83, with M = 3.03, SD = 0.62.

The Interpersonal Reactivity Index [58] is a 28-item scale. It contains four 7-item subscales; Empathic Concern (M = 3.71, SD = 0.68), Perspective Taking (M = 3.63, SD = 0.68), Fantasy (M = 3.56, SD = 0.82) and Personal Distress (M = 2.52, SD = 0.77). The items are a collection of statements (e.g., “*Sometimes I don’t feel very sorry for other people when they are having problems*”, reverse scored), for which an indication of agreement or disagreement should be provided. Each item is scored on a 5-point Likert scale from “not very well” to “very well”. The subscales showed high consistency: Empathic Concern, α = .76; Perspective Taking, α = .78; Fantasy, α = .8; Personal Distress, α = .81; full Interpersonal Reactivity Index, α = .81.

Goldberg’s 35 bipolar markers [58] were used to measure the Big Five, including Extraversion (M = 5.63, SD = 1.36, α = .83), Agreeableness (M = 6.5, SD = 1.24, α = .85), Conscientiousness (M = 6.56, SD = 1.21, α = .81), Emotional Stability (M = 5.58, SD = 1.31, α = .78), and Openness scales (M = 6.89, SD = 1.05, α = .77). Respondents rated adjectives (e.g., *Introverted (1)—(9) Extraverted*) on a 9-point Likert-type scale from 1 to 9.

### Procedure

To narrow down the context and make ‘decisions to donate’ more realistic for the participants, we chose to focus on one type of personal data—supermarket loyalty card data. Further, we identified three different potential recipients of the donated data: a famous health charity, *Cancer Research UK*; a less famous department of an academic institution, the *School of Medicine*, *University of Nottingham*, and an unspecified health research organization or charity. This was done to help contextualise participants’ decisions and to account for any organization-specific attitudes participants might hold. If the reasons for data donation are generic, we expect no differences in the structure of factors to donate personal data depending on target organisation.

Participants were randomly assigned to one of three conditions in a between-subjects design where the scenario was exactly the same: participants were asked a number of questions whether they would donate their personal data to a certain organisation, and if so, why. Each condition included a different organization: in Condition A, “Cancer”, all the questions were about Cancer Research UK, in Condition B,”MedSchool” about the School of Medicine, University of Nottingham and in Condition C, “Generic”, the questions were asked about a generic health research organization or charity. We chose Cancer Research UK and School of Medicine, University of Nottingham based on a previous study [48], where both were perceived as trustworthy, however Cancer Research UK scored much higher on recognizability than the School of Medicine, University of Nottingham. We added a generic option in Condition C to test whether the reasons to donate were independent of attitudes to particular organization.

**Fig 1:**
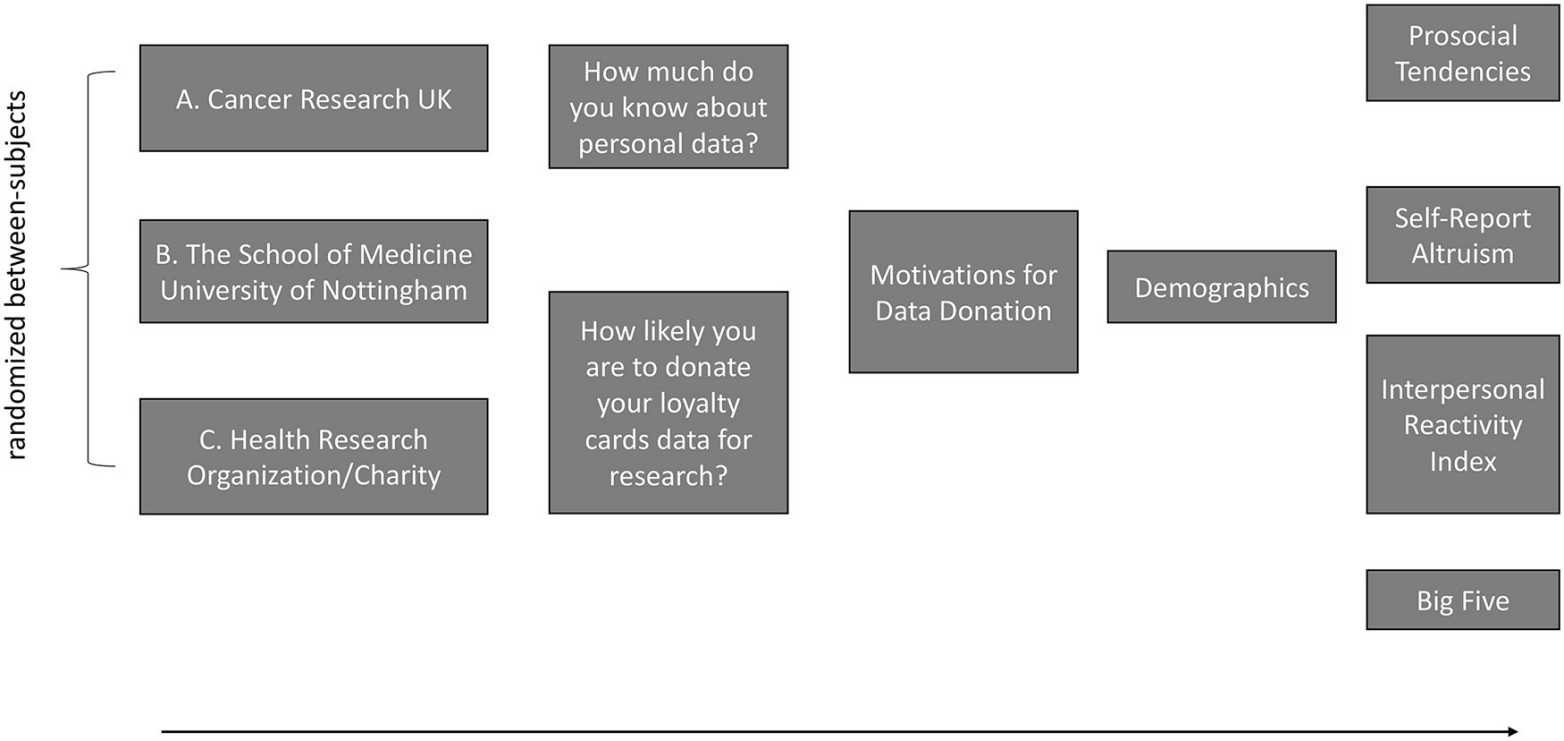
Study design.

Participants were recruited opportunistically through various accessible mailing lists of UK universities, including academic, students and non-academic mailing lists. Participants were initially presented with a consent form for the study, information about the organisation to which they had been assigned, and information explaining what data is typically collected through loyalty card usage. Once a participant had chosen to progress onto the study they were presented with questions regarding their understanding of personal data, their ownership of loyalty cards and likelihood they would donate their loyalty card data to the relevant organisation, followed by the Reasons for Data Donation questionnaire, and demographic information. Finally, participants were asked to complete Prosocial Tendencies Measure, Self-Report Altruism Scale, Interpersonal Reactivity Index and the Big Five scales. The design of the study is summarised on Fig 1.

### Participants

1,300 participants took part in the study, with 432, 432 and 436 participants being randomly assigned to Conditions Cancer, MedSchool and Generic, respectively. A close to equal number of participants dropped out from each condition after they answered the question about willingness to donate their data: 93, 90 and 87 participants from Conditions Cancer, MedSchool and Generic, respectively. The total number of participants to proceed until the end of the study were 1,030. Some of participants did not fill in Prosocial Tendencies (N = 38), Self-Report Altruism Scale (N = 44), Interpersonal Reactivity Index (N = 67) and Big Five scales (N = 79) in full. Available data was used for respective analyses. Participants were given the opportunity to be entered into a prize draw to win £50 in Amazon Vouchers. The study was approved by School of Computer Science, University of Nottingham Ethics Committee.

The majority (59.90%) of participants were male, 39.41% were female and 0.6% did not report their gender. There were no differences in gender between conditions: *x*^*2*^ (4) = 5.2, *p* = .8.

Participants ranged between 18 and 76 years of age with a mean of 27.92 (SD = 11.95). The age of participants did not vary between conditions: *F* (2,1021) = 3.04, *p* = .05. 6 participants did not report their age.

66.5% participants were born in the United Kingdom. 3 participants did not respond to this question. There was no difference in the country of birth between conditions: *x*^*2*^ (2.4) = 2, *p* = .6.

97.57% of the participants were based within the United Kingdom during their participation in the study. 3 participants did not reply to this question. There is no difference in the country of residence between conditions: *x*^*2*^ (2) = 1, *p* = .9.

41.69% of participants had no degree (1 participant did not reply to this question) and either did not study for qualifications, or finished GSCEs, A-levels or equivalent, while 58.30% completed an undergraduate degree or higher. There was a significant difference in education level between conditions: *x*^*2*^ (2) = 10.86, *p* < .01: more participants in Condition Generic (48.27%) reported no degree compared to Conditions Cancer (37.46%) and MedSchool (37.32%), while the proportion of people who reported undergraduate and higher degree was lower in Condition Generic (51.72%) compared to Condition Cancer (62.53%) and MedSchool (64.51%).

20.8% of participants worked full-time, 7.2% worked part-time, 2.6% were unemployed, retired or homemakers, and 69% were students. 4 participants indicated “Other” and 1 participant did not disclose details of their employment. There is no significant difference in employment status by condition: *x*^*2*^ (8) = 7.8, *p* = .6.

## Results

### General personal data questions

The majority of participants (67.28%, N = 1,299) reported that they understood the concept and function of personal data. 16.55% said that they did not know what personal data was and 16.16% reported that they dealt with personal data at work, while 1 participant did not respond to this question. There were no differences between conditions in terms of how much people knew about personal data: *x*^*2*^ (4) = 2.33, *p* = .5.

53.61% (Condition Cancer, 50.69%; Condition MedSchool, 49.77%; Condition Generic, 60.32%) of participants indicated that they were likely to donate their personal data with 15.31% being equally likely/unlikely to donate their data (Condition Cancer, 16.20%; Condition MedSchool, 15.51%; Condition Generic, 14.22%) and 31.08% stating that they were not likely to donate personal data (Condition Cancer, 33.10%; Condition MedSchool, 34.72%; Condition Generic, 25.46%). On average, the likelihood to donate personal data to one of three organizations, depending on condition, was more than four which was a midpoint of the scale: M = 4.42, SD = 2.08, suggesting an overall stated willingness to donate personal data. There was a significant difference in the likelihood to donate between Condition MedSchool and Condition Generic: *F* (2,1297) = 6.02, *p* < .002. Specifically, participants in Condition MedSchool reported that they were less likely to donate their data to the organization, School of Medicine, University of Nottingham (M = 4.2, SD = 2.1) in comparison to participants in Condition Generic (M = 4.68, SD = 1.95), *p* < .005. Participants in Condition Cancer (M = 4.38, SD = 2.12) were not significantly different in how likely they were to donate their personal data from Condition MedSchool or Generic.

**Fig 2:**
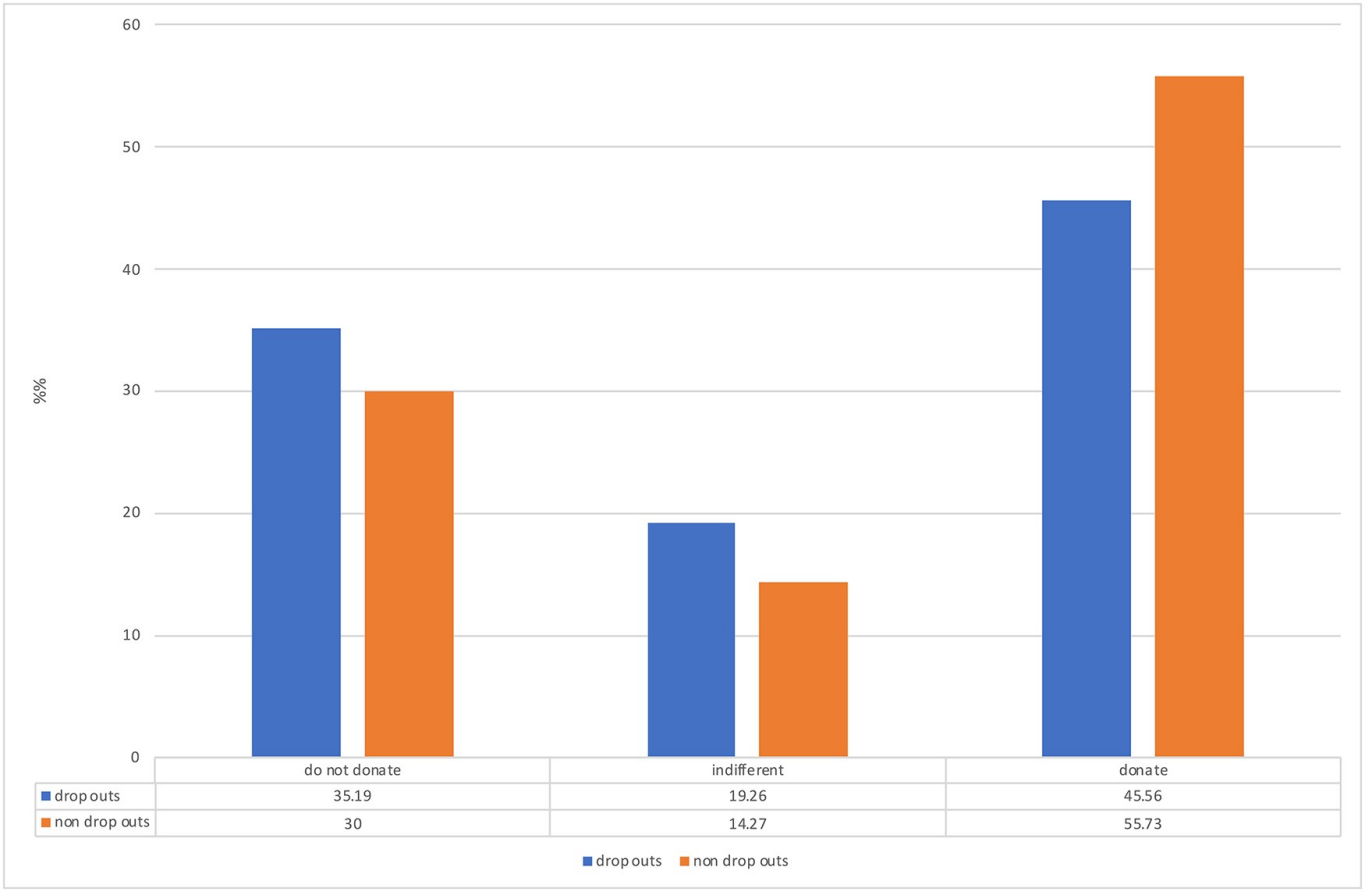
Percent of participants who were willing to donate their data, those who were indifferent and those who indicated that they would be not willing to donate, by whether they dropped out or not from the study.

We analysed whether the likelihood to donate was associated with the likelihood to drop out from the study after answering the question about likelihood to donate the data. The model was statistically significant, with *x*^*2*^ = 383.1, df = 2, *p* < .001 with an intercept of -0.97, CI+/- [-1.27:-0.67] and unstandardized beta for willingness to donate -0.09, CI+/-[-0.15:-0.02]. The Odds Ratio for willingness to donate were 1.09, CI+/- [1.02; 1.16] which means that with one unit decrease in willingness to donate personal data, the odds to drop out from the study increase by a factor of 1.09 (see Fig 2). This means that those who were less willing to donate personal data were slightly more likely to drop out from the study.

### Factor analysis of reasons for data donation

We randomly split the sample containing all three conditions into two in order to conduct exploratory and confirmatory factor analysis, with N = 515 for each of the analyses. All analyses were performed in R [66].

#### Exploratory factor analysis

The applicability of using Exploratory Factor Analysis (EFA) on this dataset was confirmed in a number of ways. All 30 items were found to correlate to at least a .3 level with at least one other item. A Kaiser-Meyer-Olkin measure of sampling adequacy was performed and was found to be .93, well above the guideline of .6; Bartlett’s test of sphericity was significant (*x*^*2*^ (435) = 8219.219, *p* < .0001). Parallel analysis on 30 items suggested three factor structure. We extracted three factors using oblique rotation. We removed eleven items that loaded less than .6 on any factor and one of pair of items which were duplicates: we kept “*If I receive a request to donate my loyalty card(s) data to Cancer Research UK*, *I would consider it a social responsibility to do so*”, we removed “*I would donate my loyalty card(s) data to Cancer Research UK because I consider it a social responsibility to do so*”. See Table 1 for factor loadings, 95% Confidence Intervals, as well as a final set of eighteen items with respective means and standard deviations. A model with a RMSEA below 0.08 indicates a good fit of the data [67]. Parallel analysis on the 18 remaining items confirmed three factors which corresponded to the predicted theoretical model, except for self-regarding and negative feelings avoidance items loading on the same factor. It explained 62% of variance with good fit statistics (based on EFA): RMSEA (95% CI) = 0.066 (0.058; 0.074). The three factors were identified as follows: (1) Social Duty to Help; (2) Purpose/Understanding; (3) Guilt/Reputation/Self-image.

**Table 1:** Reasons for Data Donation Questionnaire with factor loadings and 99% confidence intervals, EFA, as well as means (M) and standard deviations (SD) for each item.

Item	Social Duty to Help			Purpose, Understanding			Guilt, Reputation Self-image			M	SD
	Loadings	CI-	CI+	Loadings	CI-	CI+	Loadings	CI-	CI+		
1. I would donate my loyalty card(s) data to Cancer Research UK because I believe that I have a responsibility to help others.	0.74	0.63	0.84	0.11	0.05	0.17	0.08	-0.01	0.17	3.23	1.14
2. If I receive a request to donate my loyalty card(s) data to Cancer Research UK, I would consider it a social responsibility to do so.	0.82	0.72	0.90	-0.05	-0.12	0.01	-0.01	-0.10	0.09	2.79	1.14
3. When I receive a request to donate my loyalty card(s) data to Cancer Research UK, I would automatically offer my data.	0.63	0.51	0.74	-0.23	-0.30	-0.15	0.07	-0.03	0.19	2.58	1.12
4. I would donate my loyalty card(s) data to Cancer Research UK, even if no gratitude was shown in return.	0.60	0.49	0.71	0.20	0.11	0.29	-0.14	-0.24	-0.03	3.47	1.1
5. I would donate my loyalty card(s) data to Cancer Research UK because I feel that I have a duty to give back to the community.	0.71	0.59	0.81	0.05	-0.01	0.12	0.14	0.06	0.24	2.93	1.12
6. I would make the decision to donate my loyalty card(s) data to Cancer Research UK depending on the purpose of research.	0.06	-0.07	0.21	0.66	0.55	0.76	0.09	-0.03	0.19	3.91	1.05
7. I would make the decision to donate my loyalty card(s) data to Cancer Research UK based on how they would deal with my personal data.	0.18	0.07	0.28	0.72	0.63	0.81	-0.08	-0.18	0.01	3.96	1.1
8. I would make the decision to donate my loyalty card(s) data to Cancer Research UK based on how the data will be used.	-0.04	-0.12	0.04	0.86	0.79	0.92	0.00	-0.07	0.07	4.01	1.01
9. Before donating my loyalty card(s) data to Cancer Research UK, I would seek to understand the purpose of giving data for research.	-0.08	-0.19	0.02	0.78	0.69	0.86	0.06	-0.04	0.15	3.98	1.04
10. Before donating my data to Cancer Research UK, I would seek to understand how my loyalty card(s) data could help others.	-0.07	-0.18	0.04	0.71	0.61	0.80	0.08	-0.03	0.18	3.92	1.06
11. I would make a decision to donate my loyalty card(s) data to Cancer Research UK depending on what they would do with my data.	-0.03	-0.11	0.06	0.89	0.84	0.93	0.02	-0.06	0.09	4.01	1.07
12. I would make a decision to donate my loyalty card(s) data to Cancer Research UK based on who the data would be shared with.	0.11	0.01	0.21	0.77	0.69	0.85	-0.09	-0.19	0.00	4.02	1.06
13. I would donate my loyalty card(s) data to Cancer Research UK as this could relieve some guilt felt for being more fortunate than others.	0.13	0.00	0.26	-0.02	-0.10	0.07	0.59	0.46	0.73	2.05	0.98
14. Through donating my loyalty card(s) data to Cancer Research UK, I would think of those who are unlucky/ill-fated and this would help me to forget how bad I have been feeling myself.	0.01	-0.10	0.13	0.02	-0.06	0.10	0.67	0.56	0.77	2.19	1.00
15. I would donate my loyalty card(s) data to Cancer Research UK as I wish to be praised and have good reputation.	-0.02	-0.12	0.08	-0.07	-0.15	0.01	0.70	0.59	0.80	1.92	0.91
16. By donating my loyalty card(s) data to Cancer Research UK, I would be able to show people that I am a good and kind person.	0.00	-0.09	0.11	0.02	-0.06	0.10	0.76	0.66	0.85	2.18	0.92
17. If I did not donate my loyalty card(s) data to Cancer Research UK, I would feel less guilty if others did the same.	0.00	-0.14	0.15	0.12	0.02	0.22	0.60	0.47	0.73	2.41	0.94
18. By taking interest in societal issues through donation of my loyalty card(s) data to Cancer Research UK, I would feel less stressed about my own problems.	0.07	-0.04	0.20	0.00	-0.09	0.09	0.64	0.51	0.78	2.05	0.87

The *Social Duty* factor included five items referring to donation based around a social requirement or responsibility to do so; for example, ‘*I would donate my loyalty card(s) data to ORGANISATION because I consider it a social responsibility to do so*.’

The Purpose/Understanding factor included seven items which refer to a desire to know how the data would be used, for example “*I would make the decision to donate my loyalty card(s) data to ORGANISATION based on how the data will be used*”.

Finally, the *Self-Interest* factor encompassed self-regarding motives as well as avoidance of negative feelings. This can be explained by the fact that avoidance of negative feelings could be something one does because of self-interest rather than for any other reason. Its six items were focused around alleviating negative emotions (e.g., guilt), reputation and self-image. For example, “*Through donating my loyalty card(s) data to ORGANISATION*, *I would think of those who are unlucky/ill-fated and this would help me to forget how bad I have been feeling myself*.”

#### Confirmatory factor analysis

**Fig 3:**
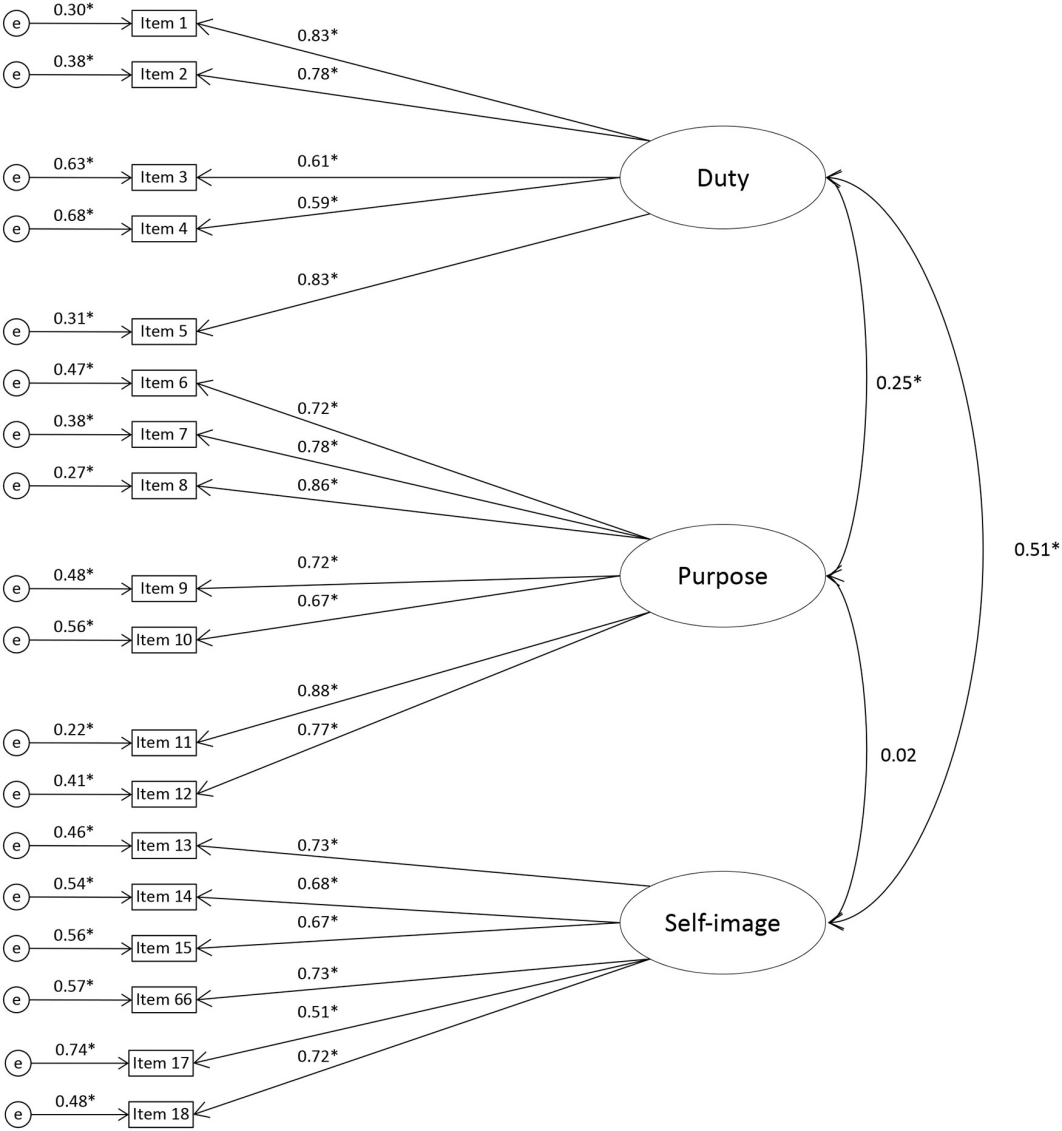
Confirmatory Factor Analysis Model with standardized estimates, Reasons for Data Donation, N = 515. *p < .001.

Confirmatory Factor Analysis (CFA) models were estimated with Sattora-Bentler correction in Psych package in R. Model fit was assessed using the *x*^*2*^-value, the root mean square error of approximation (RMSEA), the comparative fit index (CFI) and the Tucker-Lewis index (TLI). A model with a RMSEA below 0.08 and CFI and TLI greater 0.90 indicates a good fit of the data [66]. The three-factor oblique model, based on the EFA, demonstrated good fit the data: *x*^*2*^ = 435.215, df = 132, *p* < .001, RMSEA (95% CI) = 0.067 (0.060; 0.074), CFI = 0.934, TLI = 0.924, N = 515, see Fig 3. Accounting for conditions did not make a difference to the fit of the model: a configural model did not fit differently to the model with fixed loadings, *p* ns.

#### Reliability and descriptives for reasons for data donation scales

Composite scores for each scale were created by averaging the scores of items with primary loading within a single factor on overall sample, which was used for all analyses from here onwards. Each scale demonstrated high internal consistency (see Table 2 for means, standard deviations, and Cronbach’s alphas of each scales, as well as pairwise correlations between scales). The skew and kurtosis were within acceptable ranges for each subscale. All subscales were significantly correlated. There were no differences in the Reasons for Data Donation scales between conditions: Duty (*F* (1,1124) = 0.25, *p* = .5), Purpose (*F* (1,1028) = 2.25, *p* = .8), Self-image (*F* (1,1028) = 0.16, *p* = .5).

**Table 2:** Reasons for data donation subscale correlations, and means, standard deviations and Cronbach’s Alpha for subscales of Reasons for Data Donation.

	Purpose	Self-image	M (SD)	Cronbach’s Alpha
**Duty**	0.24**	0.42***	2.99 (0.88)	0.84
**Purpose**		0.07*	4.00 (0.85)	0.91
**Self-image**			2.09 (0.70)	0.83

Note:

*p < .05,

**p < .01,

***p < .001

#### Convergent validity: Reasons for data donation and other scales

The original paper [54] which have reported the questionnaire did not provide the keys to the scales. We re-ran factor analysis to investigate the factor structure of the scale. Scree plot based on factor analysis as well as parallel analysis on our data did not suggest a six-factor structure, indicating that only five factors are necessary to represent the scales.

We ran both six and five factor structure models, with the analysis yielding similar results for all items apart from Item 10: *“I believe that donating goods or money works best when it is tax-deductible*.*”* This item formed a single item factor in a six-factor structure and did not load on any factor in a five-factor structure. Therefore, we decided to remove this item for any further analysis (see S2 Table for the final five-factor structure of the scale).

Our factor composition was similar to [54] with the exception that the first factor encompassed both Public scale and items related to direct benefit from helping. We have not identified Altruism factor based on our factor composition. Cronbach’s Alphas for all scales were acceptable, ranging from .73 to .88 and are reported in S2 Table. Means and standard deviations for each Prosocial Tendencies Measure scale are reported in S2 Table.

To assess the relationship between Prosocial Tendencies Measure, Self-Report Altruism Scale and Interpersonal Reactivity Index scales and the newly created the Reasons for Data Donation scales, a series of partial correlations were performed, each partialling out two remaining Reasons for Data Donation scales, see Table 3. Generally, there were significant correlations between both the Prosocial Tendencies Measure subscales, and the Interpersonal Reactivity Index subscales, with the Reasons for Data Donation subscales in expected directions. We logged-transformed Purpose/Understanding scale for all following analyses as it was highly skewed.

**Table 3:** Correlation of each of the reasons for data donation scales, while partialling out other two scales, with Prosocial Tendencies Measure, Self-Report Altruism Scale and Interpersonal Reactivity Index.

Scales		Duty	Purpose (log)	Self-image	Willingness to donate
Prosocial Tendencies Measure (N = 992)	Public	-.08	-.13*	.47*	-.04
	Anonymous	.08	-.08	.19*	-.03
	Dire	.14*	-.01	.04	.08**
	Emotional	.06	.09	.24*	.08*
	Compliant	.19*	-.01	-.05	.11***
Self-Report Altruism Scale (N = 986)	Altruism	.14*	-.04	-.09	.04
Interpersonal Reactivity Index (N = 963)	Empathic Concern	.20*	.07	-.10	.13***
	Perspective Taking	.16*	.06	-.14*	.13***
	Fantasy	.09	.07	.05	.11***
	Personal Distress	-.05	.03	.27*	-.03
	Overall	.10	.08	.13*	.10**
Big Five (N = 951)	Extraversion	.13*	-.04	-.07	.09**
	Conscientiousness	.06	.05	-.10	.06
	Agreeableness	.14*	.02	-.10	.10**
	Emotional Stability	.04	-.04	-.07	.03
	Openness	.10	.04	-.20*	.10***

Note:

*p < .05,

**p < .01,

***p < .001

As expected, Duty correlated the highest with Empathic Concern, Perspective Taking and Complaint from Prosocial Tendencies Measure. In addition, Duty correlated with Self-Report Altruism Scale. Purpose scale correlated highest with Perspective Taking from Interpersonal Reactivity Index which also implies understanding of emotions of others, while Self-Image correlated highly with Public and Emotional Prosocial Tendencies Measure scales, as well as Personal Distress scale from Interpersonal Reactivity Index.

Finally, willingness to donate correlated positively with Dire, Emotional and Complaint Scales of Prosocial Tendencies Measure, Empathic Concern, Perspective Taking, Fantasy scales from Interpersonal Reactivity Index, as well as Extraversion, Agreeableness and Openness.

#### Reasons for data donation predicting intentions to donate personal data

Using stepwise linear regressions, we investigated how well specific dimensions of the Reasons for Data Donation questionnaire predicted willingness to donate loyalty cards data for research. Age and gender were entered first, followed by Prosocial Tendencies Measure and Self-Report Altruism Scale at Step 2, and the Reasons for Data Donation scales at Step 3. The results are reported in Table 4.

**Table 4:** Stepwise regression, with willingness to donate as an outcome and age, gender (step 1), Prosocial Tendencies Measure scales, Self-Report Altruism Scale (Step 2) and Reasons for Data Donation (Step 3). For step comparison, missing observation were deleted listwise, resulting in N = 1024.

Predictors	Step 1			Step 2			Step 3		
	B	95% CI	*ß*	B	95% CI	*ß*	B	95% CI	*ß*
Intercept	4.99***	4.62; 5.35	-0.02	4.36***	3.46; 5.21	0.03	1.30**	0.46; 2.14	0.01
Age	-0.02***	-0.03; -0.01	-0.10**	-0.2**	-0.03; -0.01	-0.11**	-0.01*	-0.02; -0.001	-0.06*
Female	0.09	-0.17; 0.36	0.05	-0.02	-0.29; 0.25	-0.01	-0.001	-0.22; 0.19	-0.01
*Prosocial Tendencies Measure*									
Public				-0.23*	-0.43; -0.02	-0.08*	-0.11	-0.22; 0.19	-0.04
Anonymous				-0.16	-0.36; 0.03	-0.06	-0.28***	-0.44; -0.13	-0.10***
Dire				0.10	-0.09; 0.27	0.04	0.02	-0.13; 0.16	0.01
Emotional				0.06	-0.11; 0.23	0.03	-0.01	-0.15; 0.13	-0.01
Compliant				0.19*	0.04; 0.34	0.09*	0.01	-0.11; 0.13	0.003
Self-Report Altruism Scale				0.14	-0.11; 0.38	0.08	-0.02	-0.20; 0.16	-0.01
*Reasons for Data Donation*									
Duty							1.6***	1.47; 1.73	0.68***
Purpose (log transformed)							0.37*	0.003; 0.74	0.05*
Self-image							-0.40***	-0.58; -0.22	-0.13***
Adjusted R2	0.01			0.03			0.42		
F for change in R2				6.71***			219.78***		

note:

* p < .05,

** p < .01,

*** p < .001

In line with predictions, Reasons for Data Donation scales added significantly to explaining variance in willingness to donate personal data: 25% of variance was explained through reasons to donate personal data, specifically Duty, Purpose and Self-Image. Duty was the strongest positive predictor of donating personal data, *ß* = 0.68, *p* < .001; while Self-Interest was a negative predictor, *ß* = -0.13, *p* < .001. Log-transformed Purpose positively predicted intentions to donate personal data, with *ß* = 0.05, *p* < .05.

## Discussion

Building on the vast prosocial behaviour and prosocial motivation literature, this study explores whether the donation of personal data could be a publicly acceptable act to support the use of consumer personal data for academic research. In a large-scale survey, we showed that over a half of participants were willing to donate personal data to research benefiting the public good. Our research adds to understanding the mechanisms of prosociality in different contexts, as well as produces a new questionnaire to measure different reasons for the donation of personal data. Similar to previous research findings [29,55] in other domains of prosocial behaviour, we showed that the strongest predictor of the decision to donate personal data was the desire to serve society, while the strongest predictor of decision not to donate personal data was the need to gain direct benefits as a result of data donation. Identifying factors why people would choose *not* to donate their data for research is also important in designing future interventions that encourage people to donate their data, as well as understanding what stops people from engaging in prosocial behaviours in general. In the case of the data donation non-motivational factors can play a role, such as privacy concerns. Future research could investigate whether and when privacy concerns could affect decisions to donate and/or if they play a moderating role on other reasons for data donation in predicting intention to donate.

In addition we identified that the need to know the consequences of donating personal data was an important third factor influencing the decision whether to donate. The donation of personal data shares similarities with other types of costly prosocial behaviours, such as blood donation, however there are also differences. When people donate blood, it is clear for what general purpose the blood will be used. When people are asked to donate data, a lot of questions about the purpose of donation can be raised. Previous research demonstrated that (un)certainty about the consequences of social actions can have effect on prosocial behaviours [68]. Personal data, such as loyalty cards data, can potentially benefit society in identifying lifestyle causes of health issues (e.g., cancer, diabetes), but it is still not clear what information can be derived from individual data that have utility for research, as well as there are risks for data misuse. Future research could investigate how the levels of certainty about the impact of data donation may influence decisions to donate personal data, and under which conditions certainty and uncertainty about the purpose of donation increases or decreases rates of donation.

We produced a new questionnaire for measuring individuals’ reasons for donating data which can be used in future research on data donation in different contexts (e.g., medical data). The newly developed questionnaire contained three scales, each providing a different insight into reasons behind data donation. *Social Duty* reflects the desire to serve society and give back to community. *Self-Interest* reflects the need to gain personal benefits as a results of data donation, such as reputation and avoiding feeling guilty. *Purpose* is a scale that reflects a need to understand the consequences of data donation as well as the importance of understanding what will be done with the data after donation. Social Duty and Self-Interest scales are in line with previously established motivations for blood donation [29]; for example, Social Duty is conceptually similar to social responsibility, and the Self-Interest scale includes elements of egalitarian warm-glow. In addition to motivational constructs discussed previously in blood donation research, we identified Purpose as a reason to donate personal data, which reflects the desire to understand the consequences of data donation. It also demonstrates the importance of the way information is presented and explained to individuals while they are making a decision to donate personal data. Our new tool—Reasons for Data Donation—showed good psychometric properties, high internal consistency as well as evidence of various forms of validity. Convergent validity was shown through significant correlations between subscales of the Reasons for Data Donation with similar subscales of the four established instruments associated with prosocial behaviour: Prosocial Tendencies Measure, Self-Report Altruism Scale, Interpersonal Reactivity Index and the Big Five. Evidence for incremental validity was demonstrated through the fact that Reasons for Data Donation scales explained the decision to donate personal data over and above domain non-specific constructs.

The major limitation of the study is that our participants only made a hypothetical decision about donating personal data. While stated intentions for actions have shown to be predictive of actual behaviour [69,70], future research could investigate whether the same reasons reflecting Social Duty, Purpose and Self-Interest predict the decision to donate when people are approached in real life. Alternatively, the percent of people who are willing to donate could depend on the qualities of the recipient organization (e.g., how trustworthy the organization is perceived to be?). In accord, we found small differences in the stated intentions to donate personal data with higher probability to donate personal data to a generic health research organization or charity than to the School of Medicine, University of Nottingham. This could be due to factors such as trust and the perceived reliability of the recipient organization. Further, the decision to donate personal data can depend on the type of data being donated: for example, Skatova *et al* [71] demonstrated that individuals are more willing to pay to protect their medical records as compared to loyalty cards data. Future research could investigate whether this in turn will affect likelihood to donate different types of their personal data for research. Another limitation of our study is that by design, those who volunteer for an online study are also more likely to be more prosocial and willing to help in general, skewing our dataset. Future studies can address this through targeted sampling of individuals from different backgrounds and with different personality characteristics.

This study, through its identification of three main reasons affecting decisions to donate personal data and its development of a questionnaire to measure those reasons, can be used to support how the need for data donation is communicated to the public. Its findings can also be used to support how the opportunities created by the use of commercial data in academic research more broadly, and health research for specifically, are communicated. Future research could study whether accounting for Social Duty, Purpose and Self-Image factors in campaigns to encourage data donation can increase the efficiency of campaigns. For example, different messages could be targeted at different groups of individuals depending on their motivational profile: e.g., for those who are high on Social Duty, the campaign could enhance benefits of data donation to the society, while for those who are high on Self-Image, the campaign framing could highlight the positive effect after sharing the act of donation on with others on social media. Motivationally targeted campaigns were shown to be more effective when encouraging prosocial behaviour in other domains [72–74]. Further, it has been shown that different forms of empathy play a role in defining various forms of prosocial motivation [61,75,76] which should make a difference in the context of data donation. Future research could investigate what personality differences or contextual factors can explain differences in reasons to donate personal data.

To summarise, the present study has shown that the data donation and reasons for data donation are similar to motivations in other subclasses of prosocial behaviour. Just over one half of respondents expressed the intention to donate their personal data, and their positive decision was mostly driven by a sense of social duty. Our study is an important step in opening up the possibilities of a novel data donation mechanism that can enable the use of commercial data for research that benefits the public. The creation and use of data generated by each and every one of us for industry is here to stay, along with all the good and bad that can entail. In these times where consumer data is mined by companies, *data donation* creates a route to redress this power imbalance by providing a safe and ethical mechanism that enables individuals to explicitly consent to what research organization they share their data with, and for what purpose.

## Supporting information

S1 Table An initial and revised list of items. (DOCX)

S2 Table Factor loadings for Prosocial Tendencies Measure. (DOCX)
