# Supplementary material for: Psychology of personal data donation
Source: PLoS One. 2019 Nov 20;14(11):e0224240. doi: 10.1371/journal.pone.0224240 (PMC6867598; doi:10.1371/journal.pone.0224240)
Supplement: S1 Table — (DOCX) [file pone.0224240.s001.docx]

#### S1 Table. An initial and revised list of items. Crosses indicate that the item was used in a particular version of the questionnaire, while ticks - that the item was retained for the final version.

| **Items** | **Initial list** | **Revised list** | **Theoretical construct** |
| --- | --- | --- | --- |
| **I would donate my loyalty card data to Charity/health organizations because I enjoy helping others in general.** | X | √ | Impure altruism |
| **If I did not donate my loyalty card data to charity/health organizations, I would feel less guilty if others did the same.** | X | X | Guilt |
| **I would donate my loyalty card data to charity/health organizations because I feel that I have to give back to the community.** | X | X | Social responsibility |
| **I would donate my loyalty card data to charity/health organizations because I believe that I have a responsibility to help others.** | X | X | Social responsibility |
| **Being upset or preoccupied would not hinder me from donating loyalty card data to charity/health organizations for public good.** | X | √ | Negative feelings |
| **I would donate my loyalty card data to charity/health organizations for public good, even though no gratitude would be shown.** | X | X | Reluctant altruism |
| **Seeing someone’s misfortune would trigger me to donate my loyalty card data to charity/health organizations for public good.** | X | √ | Negative feelings |
| **I would feel positive after donating loyalty card data to charity/health organizations for cancer research.** | X | √ | Egalitarian warm glow |
| **I would try to understand the purpose of donating loyalty card data to charity/health organizations by thinking how my personal information could help others.** | X | X | Purpose |
| **I am concerned about those who are less fortunate than me and therefore I would donate my loyalty card data to charity/health organizations to help them indirectly.** | X | √ | Social responsibility |
| **I would make the decision to donate my loyalty card data to charity/health organizations based on how it could be used for public good.** | X | X | Purpose |
| **Through donating loyalty card data to charity/health organizations, I would feel better about myself.** | X | √ | Egalitarian warm glow |
| **By taking interest in societal issues through donating loyalty card data to charity/health organizations, I would feel less stressed about my own problems.** | X | X | Negative feelings |
| **Through donating loyalty card data to charity/health organizations, I would feel good for being appreciated (e.g. receiving a thank you note).** | X | √ | Self-regarding |
| **I would donate my loyalty card data to charity/health organizations as I wish I could be praised and have good reputation.** | X | X | Self-regarding |
| **Through donating loyalty card data to charity/health organizations, I would think of those who are unlucky/ill-fated and this helps me to forget how bad I have been feeling.** | X | X | Negative feelings |
| **I would donate my loyalty card data to charity/health organizations as this could relieve some of the guilt I felt for being more fortunate than others.** | X | X | Guilt |
| **Donating loyalty card data to charity/health organizations to help others would increase my self-esteem.** | X | √ | Egalitarian warm glow |
| **Through donating loyalty card data to charity/health organizations, I could show people that I am a good and kind person.** | X | X | Egalitarian warm glow |
| **When I receive a request to donate loyalty card data to charity/health organizations, I would automatically offer my loyalty card data.** | X | X | Social responsibility |
| **I think my loyalty card data donated to charity/health organizations would be capable to help others.** | X | √ | Purpose |
| **I would make the decision to donate my loyalty card data to charity/health organizations based on how charity/health organizations deal with personal information.** | X | X | Purpose |
| **I would donate my loyalty card data to charity/health organizations because I could obtain tokens in return (e.g. gift voucher).** | X | √ | Self-regarding |
| **I would make the decision to donate my loyalty card(s) data to Cancer Research UK depending on the purpose of research.** | X | X | Purpose |
| **If I receive a request to donate my loyalty card(s) data to Cancer Research UK, I would consider it a social responsibility to do so.** | X | X | Social responsibility |
| **If I donate my loyalty card(s) data to Cancer Research UK I would like to receive a report on the study my data was used for.** | X | √ | Purpose |
| **Before donating my data to Cancer Research UK, I would seek to understand how my loyalty card(s) data could help others.** | X | X | Purpose |
| **I would make a decision to donate my loyalty card(s) data to Cancer Research UK depending on what they would do with my data.** | X | X | Purpose |
| **I would make a decision to donate my loyalty card(s) data to Cancer Research UK based on who the data would be shared with.** | X | X | Purpose |
| **I would donate my loyalty card(s) data to Cancer Research UK because I consider it a social responsibility to do so.** | X | √ | Social responsibility |
