# Supplementary material for: Psychology of personal data donation
Source: PLoS One. 2019 Nov 20;14(11):e0224240. doi: 10.1371/journal.pone.0224240 (PMC6867598; doi:10.1371/journal.pone.0224240)
Supplement: S2 Table — (DOCX) [file pone.0224240.s002.docx]

#### S2 Table. Factor loadings for Prosocial Tendencies measure. The loadings below .3 are suppressed. Loadings in bold indicate which factor the item belongs to.

| Items | Public/  direct benefit | Anonymous | Emotional | Dire | Compliant |
| --- | --- | --- | --- | --- | --- |
| 1. I can help others best when people are watching me. | **0.74** |  |  |  |  |
| 3. When other people are around, it is easier for me to help needy others. | **0.60** |  |  |  |  |
| 4. I think that one of the best things about helping others is that it makes me look good. | **0.74** |  |  |  |  |
| 5. I get the most out of helping others when it is done in front of others. | **0.79** |  |  |  |  |
| 13. Helping others when I am in the spotlight is when I work best. | **0.75** |  |  |  |  |
| 16. I believe I should receive more recognition for the time and energy I spend on charity work. | **0.68** |  |  |  |  |
| 20. One of the best things about doing charity work is that it looks good on my resume. | **0.65** |  |  |  |  |
| 23. I feel that if I help someone, they should help me in the future. | **0.52** |  |  |  |  |
| 8. I prefer to donate money anonymously. |  | **0.55** |  |  |  |
| 11. I tend to help needy others most when they do not know who helped them. |  | **0.75** |  |  |  |
| 15. Most of the time, I help others when they do not know who helped them. |  | **0.73** |  |  |  |
| 19. I think that helping others without them knowing is the best type of situation. |  | **0.66** |  |  |  |
| 22. I often make anonymous donations because they make me feel good. |  | **0.49** |  |  |  |
| 12. I tend to help others particularly when they are emotionally distressed. |  |  | **0.62** | 0.45 |  |
| 17. I respond to helping others best when the situation is highly emotional. |  |  | **0.64** |  |  |
| 21. Emotional situations make me want to help needy others. |  |  | **0.71** |  |  |
| 2. It is most fulfilling to me when I can comfort someone who is very distressed. |  |  | **0.53** | 0.31 |  |
| 6. I tend to help people who are in real crisis or need. |  |  | 0.31 | **0.60** |  |
| 9. I tend to help people who hurt themselves badly. |  |  |  | **0.52** |  |
| 14. It is easy for me to help others when they are in a dire situation. |  |  |  | **0.62** |  |
| 7. When people ask me to help them, I don’t hesitate. |  |  |  |  | **0.91** |
| 18. I never hesitate to help others when they ask for it. |  |  |  |  | **0.72** |
| M | 1.71 | 2.56 | 2.99 | 3.10 | 3.55 |
| SD | 0.71 | 0.73 | 0.94 | 0.91 | 0.94 |
| Alpha | .88 | .73 | .80 | .72 | .84 |
